# Distinguishing Nontuberculous Mycobacteria from Multidrug-Resistant *Mycobacterium tuberculosis*, China

**DOI:** 10.3201/eid2006.130700

**Published:** 2014-06

**Authors:** Kaijin Xu, Sheng Bi, Zhongkang Ji, Haiyang Hu, Feisu Hu, Beiwen Zheng, Bing Wang, Jingjing Ren, Shigui Yang, Min Deng, Ping Chen, Bing Ruan, Jifang Sheng, Lanjuan Li

**Affiliations:** State Key Laboratory for Diagnosis and Treatment of Infectious Diseases, the First Affiliated Hospital, School of Medicine, Zhejiang University, Hangzhou, China (K. Xu, S. Bi, Z. Ji, H. Hu, F. Hu, B. Zheng, B. Wang, J. Ren, S. Yang, M. Deng, P. Chen, B. Ruan, J. Sheng, L. Li);; Collaborative Innovation Center for Diagnosis and Treatment of Infectious Diseases, Hangzhou (K. Xu, L. Li)

**Keywords:** tuberculosis, Mycobacterium tuberculosis, nontuberculous Mycobacteria, multidrug-resistant Mycobacterium tuberculosis, China, tuberculosis and other mycobacteria, MDR TB

**To the Editor:** Mycobacteria are commonly characterized by positive acid-fast staining. Most mycobacterial species belong to the nontuberculous mycobacteria (NTM), excluding species in the *Mycobacterium tuberculosis* complex and *M. leprae*. Both *M. tuberculosis* and NTM can induce pulmonary infection with similar symptoms and pulmonary radiographic findings (*1*). These similarities have led to difficulty in distinguishing these infections clinically.

As in many developing countries, the acid-fast stain is the only bacteriologic basis for diagnosing tuberculosis (TB) in primary health care institutions in China, where facilities are limited for *M. tuberculosis* culture, strain identification, and drug resistance detection. Thus, NTM is easily misdiagnosed as *M. tuberculosis*, and multidrug-resistant (MDR) TB is unable to be accurately identified. Patients with misdiagnosed TB usually are treated with the standard anti-TB regimens recommended by the Chinese government (i.e., 2HRZE/4HR [2 months of isoniazid (INH), rifampin (RIF), pyrazinmid, and ethambutol, followed by 4 months of INH and RIF 1 time daily] and 2H_3_R_3_Z_3_E_3_/4H_3_R_3_ [2 months of INH, RIF, pyrazinamide, and ethambutol followed by 4 months of INH and RIF 3 times weekly]) (*2*), which often results in treatment failures. Misdiagnosis is a key hurdle for effective prevention and treatment of TB (*3*–*5*). To evaluate the effect of misdiagnosis on TB prevention, we determined the proportion of patients with MDR TB and NTM infection in primary health care institutions in Zhejiang Province, China. Our findings would be useful for improving TB prevention and treatment.

During 2011–2012, sputum samples from 13,882 patients suspected of having TB in 8 counties in Zhejiang Province were used to culture mycobacteria. Each sample was seeded onto 2 pieces of Löwenstein-Jensen medium. A total of 1,410 samples grew mycobacteria confirmed as acid-fast bacilli by using Ziehl-Neelsen staining. The 1,410 samples were further identified by using the Mycobacteria Identification Array Kit (CapitalBio, Beijing, China). The kit contains 17 types of bacilli-specific 16S rRNA probes (i.e., *M. tuberculosis* complex, *M. avium*, *M. intracellulare*, *M. gilvum*, *M. xenopi*, *M. smegmatis*, *M. aurum*, *M. terrae*, *M. gordonae*, *M. chelonae/abscessus*, *M. phlei*, *M. scrofulaceum*, *M. fortuitum*, *M. szulgai*, *M. ulcerans*, *M. marinum*, and *M. kansasii*). With this method, *M. tuberculosis* and NTM can be distinguished, and the species of NTM can be identified (*6*–*8*). Of 1,410 positive strains, we identified 1,332 (94.5%) as *M. tuberculosis* and 78 (5.5%) as NTM. NTM strains were further identified as follows: *M. intracellulare*, 39 isolates; *M. chelonae/abscessus*, 12 isolates, *M. kansasii*, 13 isolates; *M. avium*, 3 isolates; *M. fortuitum*, 4 isolates; and *M. scrofulaceum* and *M. szulgai*, 1 isolate each. For 5 isolates, strain could not be classified.

We detected drug resistance of 1,332 *M. tuberculosis* strains using a Tuberculosis Drug Resistance Detection Array Kit (CapitalBio) (*9*). The mutant points for RIF resistance were identified as follows: *rpoB*/C531G, C531T, CG531AC, A526C, A526G, A526T, C526A, C526G, C526T, T533C, A516G, A516T, G516T, T511C, T511G, C513A, A513T, and C522T (Table). Moreover, the kit contained 5 mutant points for INH resistance, including *katG* (G315A, G315C, G315T, and C315) and *inhA* (C-15T) (Table). Of 1,332 *M. tuberculosis* strains, we identified 1,115 (83.7%) RIF/INH-sensitive strains, 88 (6.6%) MDR TB strains, 83 (6.2%) INH-resistant strains, and 47 (3.5%) RIF-resistant strains. Of the 1,410 positive strains, 88 (6.2%) were MDR *M. tuberculosis* strains.

**Table:** Gene mutations of 214 drug-resistant tubercle bacilli, Zhejiang Province, China, 2011–2012

Drug	Mutant points	Mutant times		
Mutant times for single site, no. (%)*	Total no. mutant times of sites related to drug resistance	
Isoniazid	*inhA-*15 (C→T)	32 (18.6)	172†	
	*katG*315 (G→C), (G→A)	140 (81.4)		
Rifampin	*rpoB*511 (T→C)	10 (7.1)	140‡	
	*rpoB*513 (A→C)	2 (1.4)		
	*rpoB*516 (A→T), (A→G), (G→T)	19 (13.6)		
	*rpoB*526 (A→G), (A→T), (C→G), (C→T)	21 (15)		
	*rpoB*531 (C→G), (C→T)	84 (60.0)		
	*rpoB*533 (T→C)	4 (2.9)		

*No. mutant times for single site/total no. mutant times of sites related to drug resistance. †A strain simultaneously had *katG*315 (G→C) and *inhA*-15 (C→T). ‡Five strains had the double mutation of *rpoB*.

The epidemiology of TB in Zhejiang Province reflects the situation in China and some developing countries (*10*). The clinical diagnosis and treatment of >80% TB cases in China is performed mainly by primary health care institutions. However, almost 80% of these medical institutions do not have the capability to culture *M. tuberculosis*, detect drug resistance, and identify strains (*7*). Of 1,410 strains obtained from the patients in 8 counties of Zhejiang Province, 218 (15.5%) were MDR TB, INH resistant, and RIF resistant. These affected patients could not be effectively treated with the national standard regimens. Specifically, 88 patients with MDR TB would be at risk for extensively drug-resistant TB, and 83 patients with INH-resistant TB and 47 with RIF-resistant TB would be at risk for MDR TB. In addition, we identified 78 (5.5%) NTM strains. With the acid-fast stain, these illnesses would be misidentified as TB and, in most instances, also would be reported as treatment failures. Clearly, accurate diagnosis provided by the technologies used in this study for distinguishing NTM and *M. tuberculosis*, *Mycobacterium* strain identification, and drug-resistance detection would increase the cure rate and effectively prevent TB epidemics.

For INH resistance, *katG315* was a main mutant point of the *M. tuberculosis* strain; 140 (81.4%) of the 172 INH-resistant mutations were related to *katG315*. For RIF resistance, *rpoB531* was a main mutant point; 84 (60.0%) of 140 RIF-resistant mutations were associated with *rpoB531*. Therefore, in future studies, more attention should be paid to the molecular epidemiology of *katG315* and *rpoB531*. 

In conclusion, using the techniques for *M. tuberculosis* culture, *Mycobacterium* strain identification, and drug-resistance detection is necessary. It should be urgently pursued for accurate TB diagnosis in primary health care institutions in China to improve the prevention, treatment, and control of TB.
